# Enhancing flood prediction through physics-driven typhoon feature engineering and machine learning

**DOI:** 10.1371/journal.pone.0346237

**Published:** 2026-04-20

**Authors:** Zhi Zhang, Yusha Xiao, Biqing Chen, Kaihao Long, Feng Liang, Jiwu Liao

**Affiliations:** 1 Tourism and Historical Culture College, Zhaoqing University, Zhaoqing, China; 2 School of Computer Science and Software, Zhaoqing University, Zhaoqing, China; 3 School of Geography and Planning, Sun Yat-sen University, Guangzhou, China; Swedish Meteorological and Hydrological Institute, SWEDEN

## Abstract

Typhoon-induced extreme floods pose severe threats to subtropical watersheds, yet systematic integration of typhoon physics into machine learning flood prediction remains limited. This study developed a physics-informed machine learning framework for the Boluo watershed, South China, emphasizing typhoon feature engineering. Four models (Linear Regression (LR), Artificial Neural Network (ANN), Random Forest (RF) and XGBoost (XGB)) were systematically evaluated across three feature engineering scenarios: Baseline (conventional hydrometeorological variables), With Typhoon (original typhoon observations), and Enhanced Typhoon (19 physics-informed derived features). Physics-driven design included sigmoid-transformed distance decay functions representing saturating near-field typhoon influence, multi-day cumulative impact indices integrating antecedent storm effects, and trajectory-based kinematic features characterizing translation speed and directional evolution. ANN-EnTY achieved superior performance with Kling-Gupta Efficiency (KGE) of 0.946 and Root Mean Square Error (RMSE) of 174 m³/s, representing a 3.1% improvement in KGE and 16.7 m³/s reduction in RMSE compared to the Baseline scenario. During a representative extreme flood event with peak flow of 7670 m³/s, ANN-EnTY reduced peak prediction error by approximately 4% relative to the best-performing baseline model. SHAP analysis revealed upstream flow dominance (72.6%), while typhoon features, contributing only 2% overall, played critical synergistic roles during extremes. This dual-mode pattern of routine memory-driven versus extreme event-driven responses provides mechanistic insights for operational flood warning systems. The framework offers replicable methodology for typhoon-prone watersheds with direct implications for disaster preparedness and water management.

## 1. Introduction

Accurate streamflow prediction remains fundamental to water resources management, flood early warning systems, reservoir operations, and ecological flow protection. Climate change and intensifying extreme weather events have substantially increased the frequency and magnitude of catastrophic floods, underscoring the critical need for reliable forecasting tools to support disaster mitigation and sustainable water management [1–2]. Among extreme events, tropical cyclones pose unique challenges to hydrological forecasting due to their intense precipitation, complex spatial-temporal dynamics, and devastating flood impacts [3–4]. In subtropical monsoon regions such as South China, typhoon-induced extreme floods constitute a primary hydrological hazard, yet systematic integration of typhoon characteristics into machine learning-based flood prediction frameworks remains underexplored.

Traditional streamflow forecasting approaches broadly encompass process-based hydrological models and data-driven machine learning methods. Process-based models such as Hydrologic Engineering Center – Hydrologic Modeling System (HEC-HMS) and Soil and Water Assessment Tool (SWAT) simulate rainfall-runoff processes through explicit representation of physical hydrological mechanisms, providing mechanistic understanding of watershed behavior [5]. However, these models typically require extensive input data including detailed watershed characteristics, soil properties, and land use information that are often unavailable in data-scarce regions. Moreover, process-based models frequently struggle to capture the highly nonlinear and nonstationary patterns inherent in typhoon-driven hydrological processes, particularly in watersheds influenced by complex meteorological forcing and reservoir operations [6].

In contrast, machine learning approaches have demonstrated remarkable capability in extracting complex patterns from high-dimensional data without requiring explicit physical parameterization. Recent advances in deep learning, particularly Long Short-Term Memory networks, have shown superior performance in capturing complex hydrological dynamics, with studies demonstrating their effectiveness in predicting extreme events even when comparable extremes are not included in training datasets [7]. Artificial Neural Networks have proven effective in rainfall-runoff modeling through their hierarchical feature learning capacity, while ensemble tree-based methods including RF, Extra Trees, and XGB have achieved high accuracy in streamflow prediction, with ensemble methods accounting for nearly 80% of best-performing models in comparative evaluations [8–9].

Recent paradigm shifts toward physics-informed machine learning have sought to synergistically combine the predictive power of data-driven models with physical constraints governing hydrological processes. Hybrid approaches that integrate process-based model outputs with deep learning have demonstrated substantial improvements in streamflow prediction, particularly by reducing uncertainty and enhancing peak flow timing accuracy [10–11]. Studies have shown that incorporating physically-based inferences significantly improves model performance compared to purely data-driven approaches, especially in watersheds subject to human interventions such as dam operations [12]. Multi-dimensional feature engineering frameworks that systematically combine meteorological forcing variables, topographical characteristics, soil moisture observations, and land surface properties have shown particular promise for extreme event prediction. For instance, Wang et al. [13] integrated multiple sources of information including Hydrology-related data from the Global Land Data Assimilation System (GLDAS), hydro-meteorological and streamflow data, to better capture the complexity of the hydrological processes. The integration of glacio-hydrological model outputs with deep learning architectures has further demonstrated the value of embedding physical process understanding into machine learning frameworks [14].

Typhoon feature engineering for flood prediction encompasses three primary methodological approaches. Statistical feature extraction derives aggregate metrics (mean, maximum, standard deviation) from raw typhoon observations, offering computational efficiency but lacking physical interpretability and failing to capture nonlinear spatial-temporal dynamics [15]. Deep learning-based automatic feature extraction through convolutional neural networks or attention mechanisms demonstrates impressive performance in data-rich contexts [16], yet requires extensive training datasets typically unavailable for rare extreme events and offers limited operational interpretability [17]. Physically derived feature engineering constructs features based on tropical cyclone physics, including nonlinear distance decay functions, trajectory-based kinematic features, and cumulative temporal indices, achieving optimal balance between process representation and data efficiency while enabling effective generalization to unseen extreme events and maintaining interpretability for operational forecasting [18–19].

Despite growing applications of machine learning in hydrology, critical research gaps persist in typhoon-induced flood prediction. Systematic integration of typhoon characteristics into streamflow forecasting frameworks remains limited, with most studies treating typhoons merely as precipitation sources rather than explicitly modeling their dynamic physical properties such as intensity evolution, translation speed, and distance decay effects. Extreme flood events, typically defined as flows exceeding the 90th percentile, constitute only approximately 10% of observational records in hydrological datasets [20–21], creating severe class imbalance during model training that systematically biases predictions toward prevalent low-flow regimes. Recent studies have demonstrated that conventional machine learning models frequently underestimate extreme flood peaks, with underestimation magnitudes ranging from 10% to over 30% depending on model architecture and training strategies [22–23]. Models trained on imbalanced datasets may fail to capture the full magnitude of rare but high-consequence events [7], underscoring the urgent need for targeted methodological innovations addressing extreme value prediction in typhoon-influenced watersheds. Recent advances in tropical cyclone modeling have demonstrated the potential of hybrid physics-ML approaches for track and intensity forecasting [24], yet translation of these methodologies to watershed-scale flood prediction remains nascent. The challenge of systematic peak underestimation in machine learning models for extreme flood events has been widely documented [25].

Moreover, explainable artificial intelligence techniques have emerged as powerful tools for unveiling hydrological process mechanisms, with SHAP analysis enabling interpretation of feature contributions and interactions in flood prediction models [26–27]. Recent studies have demonstrated that SHAP-based interpretability can reveal dynamic flood drivers and their temporal evolution, identifying shifts between routine flow dependence on antecedent conditions versus extreme event dependence on meteorological forcing [28–29]. However, critical limitations of SHAP in hydrological applications warrant consideration. The additive assumption underlying SHAP may inadequately capture complex nonlinear feature interactions during extreme events [30]. Furthermore, SHAP interpretations can be misleading when input features exhibit high multicollinearity [31]. Despite these limitations, systematic application of explainable AI to disentangle watershed memory effects versus typhoon forcing during extreme events remains limited, particularly in quantifying synergistic roles of physically-derived features and revealing dual-mode prediction mechanisms governing routine versus extreme flood generation.

**Fig 1:**
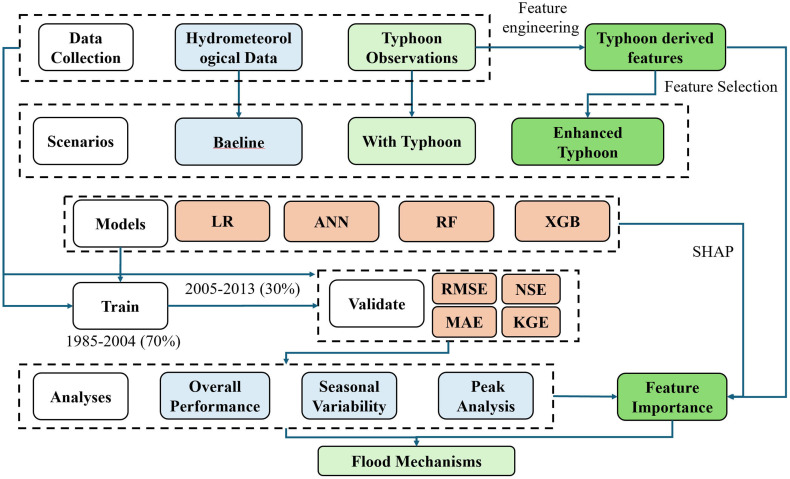
The workflow of this study.

Therefore, this study aims to develop and evaluate an integrated physics-informed machine learning framework for typhoon-induced flood prediction in the Boluo watershed, South China, with emphasis on extreme event forecasting through strategic feature engineering and targeted model optimization. The specific objectives are to: (1) Systematically compare machine learning models (LR, ANN, RF, XGB) across baseline, typhoon-included, and physics-enhanced feature scenarios to quantify the predictive value of typhoon feature engineering; (2) Apply SHAP-based explainable AI to quantify feature importance and reveal the dual-mode mechanism of routine memory-driven versus extreme event-driven flood generation. The core innovation lies in systematically transforming raw observed typhoon data (direct measurements of position, wind speed, and pressure at discrete time points) into physically derived features that explicitly encode tropical cyclone dynamics governing watershed flood response. This explicit physics encoding reduces model dependency on extensive training datasets, enhances interpretability by aligning features with known atmospheric-hydrological processes, and improves generalization capability to rare extreme events by incorporating fundamental tropical cyclone dynamics that transcend specific historical realizations. Distinct from conventional approaches relying solely on hydrological monitoring, this study integrates physics-informed typhoon features to reveal the dual-mode prediction mechanism governing extreme floods, providing explicit mechanistic guidance for optimizing operational flood early warning systems. This comprehensive framework bridges the gap between atmospheric typhoon dynamics and watershed hydrological response, advancing physics-informed machine learning for operational flood forecasting in typhoon-prone subtropical monsoon watersheds. By explicitly modeling typhoon physics through derived features, this study addresses critical operational needs for reliable flood prediction during high-consequence scenarios while providing interpretable insights into underlying hydrological mechanisms. The workflow of this study is shown as Fig 1.

## 2. Materials and methods

### 2.1. Study area and dataset

**Fig 2:**
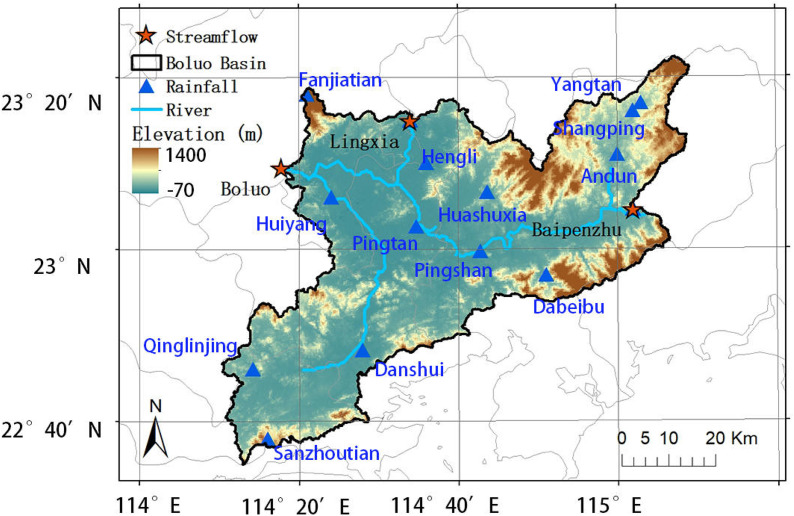
Location and topographic characteristics of the Boluo watershed in the Dongjiang River Basin, southern China. Digital Elevation Model (DEM) data were obtained from the Shuttle Radar Topography Mission (SRTM) at 90-meter resolution. Administrative boundary data were obtained from the Standard Map Service of the Ministry of Natural Resources of China (http://bzdt.ch.mnr.gov.cn/). All these datasets are publicly accessible (S1 File).

This study focuses on the Boluo watershed, a strategically important sub-basin of the Dongjiang River system within the Pearl River Basin, South China (Fig 2). Located in a typhoon-prone coastal region, the watershed experiences frequent tropical cyclone impacts during the annual typhoon season (June to October), with historical records indicating multiple extreme flood events triggered by landfalling or nearby-passing typhoons. Originating in Jiangxi Province and flowing southwest through Guangdong Province, the Dongjiang River serves as a vital water source for the Guangdong-Hong Kong-Macao Greater Bay Area, making accurate typhoon-flood prediction critically important for regional water security and disaster mitigation.

The Boluo watershed, positioned as the outlet basin of the Dongjiang River system, encompasses a drainage area of 3941.3 km² with a shape ratio of 1.06, indicating a relatively compact configuration that facilitates rapid runoff concentration during intense typhoon rainfall events. The watershed’s hydrological response is substantially governed by upstream flow contributions from the Lingxia gauge station and the Baipenzhu Reservoir, the latter being a key flood control infrastructure with regulated storage operations that significantly modulate downstream flood dynamics.

### 2.2. Dataset

The modeling framework integrates multi-source hydrometeorological observations and tropical cyclone track data to predict daily streamflow at the Boluo outlet, with particular emphasis on capturing the spatially heterogeneous and temporally dynamic characteristics of typhoon-induced floods. Precipitation measurements were collected from 14 rain gauge stations across diverse topographical zones (mountainous terrain, river plains, and valley corridors). Complementary basin-averaged rainfall and evapotranspiration estimates were derived from national meteorological control stations to provide large-scale atmospheric forcing context. Streamflow observations encompassed daily discharge records from three hydrological gauges (Boluo, Lingxia, and Baipenzhu), collectively capturing the watershed’s runoff generation, channel routing, and reservoir regulation dynamics.

Typhoon track and intensity data were obtained from the International Best Track Archive for Climate Stewardship (IBTrACS), the most comprehensive global tropical cyclone database maintained by NOAA’s National Centers for Environmental Information. IBTrACS provides 6-hourly observations of tropical cyclone characteristics including geographical position (latitude and longitude), maximum sustained wind speed, minimum central pressure, and storm classification. For this study, all typhoon events affecting the Boluo watershed during 1985–2013 were extracted based on a 500 km influence radius criterion, yielding a comprehensive catalogue of tropical cyclone impacts ranging from distant peripheral effects to direct landfalls. Raw 6-hourly IBTrACS records were temporally interpolated to daily resolution to align with hydrological observations, preserving the dynamic evolution of typhoon trajectories and intensity variations.

From these foundational observations, 19 physics-informed typhoon features were systematically engineered across six categories: (1) Binary state and intensity encoding (typhoon activity flag, intensity classification, wind force level); (2) Physics-constrained spatial influence metrics (sigmoid-transformed distance decay function, comprehensive impact index integrating wind speed, pressure, and proximity, standardized pressure intensity); (3) Temporal persistence descriptors (typhoon duration days); (4) Multi-scale cumulative effects capturing antecedent typhoon influences through rolling window aggregations (3-day and 7-day cumulative activity, 3-day and 7-day cumulative intensity); (5) Kinematic trajectory features characterizing typhoon movement patterns (longitudinal and latitudinal change rates, translation speed, relative longitude and latitude positions with respect to watershed centroid); and (6) Seasonal modulation features accounting for monsoon climate variability (month indicator, typhoon season flag, season-intensity interaction term). The distinction between observed typhoon features and physically derived features warrants emphasis. Observed features comprise direct measurements from meteorological monitoring systems that capture instantaneous tropical cyclone state but do not explicitly represent physical mechanisms governing typhoon-watershed interactions. In contrast, physically derived features transform raw measurements into engineered quantities encoding tropical cyclone physics. This transformation from observations to physics-informed features enables machine learning models to leverage domain knowledge rather than discovering complex relationships solely from limited historical extreme events.

Rigorous data quality control procedures were implemented to ensure reliability for typhoon event modeling. Missing daily observations, accounting for less than 3% of the total record, were imputed using linear interpolation for short gaps (under five consecutive days) where temporal continuity and smooth transitions are physically reasonable based on the high autocorrelation structure of hydrological processes. Extended data gaps were addressed by substituting climatological means computed from corresponding calendar periods and hydrological regime classifications (wet, normal, dry years) to preserve seasonal patterns and magnitude distributions while avoiding bias toward overall mean values. Outliers were identified using the three-sigma criterion and replaced with centered moving averages from adjacent normal observations to preserve temporal continuity while eliminating erroneous measurements. For handling missing observations in hydrometeorological datasets, various imputation techniques exist, including k-nearest neighbor (kNN) imputation, and multiple imputation by chained equations (MICE). In this study, we selected a simple statistical-based imputation due to their computational efficiency, straightforward interpretability, and widespread adoption in operational hydrological forecasting systems. While advanced methods like kNN and MICE offer sophisticated multivariate imputation capabilities, they require careful parameter tuning (kNN’s distance metrics and neighbor count) and substantial computational resources (MICE’s iterative procedures), which may limit their applicability.

Accounting for heterogeneous observation record lengths across monitoring stations, the complete dataset (1985–2013) was partitioned into training (1985–2004, comprising 70% of the record) and independent testing phases (2005–2013, 30% holdout). This temporal split ensures both subsets encompass diverse hydrological conditions, including multiple typhoon seasons with varying intensity distributions, thereby enhancing model robustness for extreme event prediction. It should be noted that the train-test splitting was employed without implementing specialized data imbalance handling techniques, as the primary focus was placed on evaluating the contribution of physics-informed typhoon feature engineering to extreme flood prediction within standard machine learning frameworks. The training phase was used for both hyperparameter optimization via 5-fold timeseries split cross-validation and final model training with optimal hyperparameters, while the testing period provided unbiased assessment of predictive skill and generalization capacity to unseen typhoon events. All performance metrics reported in the Results section are calculated on this independent test set.

### 2.3. Feature selection and sensitivity analysis

To identify the most informative typhoon features and eliminate redundancy, two complementary feature selection methods Recursive Feature Elimination (RFE) and Mutual Information (MI) were employed.

RFE is a wrapper-based backward selection method that recursively removes the least important features. Given a training dataset containing *N* samples (each sample consists of a feature vector *x*_*i*_ and a corresponding target value *y*_*i*_) with n features in total, RFE iteratively: (1) Trains a base estimator (linear regression in this study) on the current feature set *F*_*k*_; (2) Ranks features by their coefficients; (3) Removes the lowest-ranked feature(s); (4) Repeats until the desired number of features m is reached. RFE was configured to select 50 features with a step size of 10.

MI measures the statistical dependence between each feature (*X*_*j*_) and the target variable (*Y*), quantifying the reduction in uncertainty about *Y* when *X*_*j*_ is known. The core formula is:


$$\begin{array}{c} \text{M}I\left(X_{j},\;Y\right)=\;\iint \text{p}\left(x_{j},\;y\right)\;\times \text{log }[\frac{\text{p}\left(x_{j},\;y\right)}{\text{p}\left(x_{j}\right)\times \text{p}(y)}]\;{\text{d}x}_{j}\text{d}y \end{array}$$ (1)

Where $p\left(x_{j},\;y\right)$ is the joint probability of *X*_*j*_ and *Y* occurring simultaneously; $p\left(x_{j}\right)$ and p(y) are the marginal probabilities of *X*_*j*_ and *Y* respectively. For regression tasks, MI is estimated using k-nearest neighbors (*k* = 3 in this study). Features with higher MI scores capture more information about the target. The top 50 features ranked by MI were selected for comparison with RFE.

### 2.4. Models

Four machine learning models were employed for typhoon-induced streamflow prediction: LR, ANN, RF, XGB. All models underwent systematic hyperparameter optimization using GridSearchCV with TimeSeriesSplit cross-validation (5 folds) to preserve temporal ordering and prevent data leakage. After selecting optimal hyperparameters through cross-validation, final models were retrained on the complete training period (1985–2004) and evaluated on the independent test period (2005–2013). Hyperparameter search spaces were designed to balance model complexity and computational efficiency, with optimization targeting minimum validation RMSE.

LR establishes linear input-output relationships through ordinary least squares optimization [32], serving as an interpretable baseline despite its simplifying linearity assumption. Hyperparameter tuning examined fit_intercept [True, False], positive constraint [True, False] to ensure physically plausible non-negative streamflow predictions, and regularization strategy [None, Ridge with alpha=(0.1, 1.0, 10.0)] to prevent overfitting in high-dimensional feature spaces. The optimal LR configuration (fit_intercept = ’True,’ positive constraint = ’True,’ regularization strategy = Ridge with alpha 0.1) was selected.

ANN employs multi-layer perceptron architecture with ReLU activation functions for hidden layers, enabling universal approximation of complex typhoon-rainfall-runoff transformations [33]. Architecture search explored hidden layer configurations [(50,), (100,), (50, 50)], activation functions (ReLU, tanh), and learning rate strategies (constant versus adaptive). Regularization through early stopping (patience = 100 epochs) and dropout (rate = 0.2) prevented overfitting on limited extreme event samples. The optimal configuration (100 hidden units, ReLU activation, adaptive learning) was selected based on flood period validation performance. The ANN optimal configuration (hidden_layer_sizes=(100,), activation = ’relu,’ solver = ’adam,’ learning_rate = ’adaptive,’ alpha = 0.0001) was selected based on flood period validation performance.

RF constructs decision tree ensembles trained on bootstrapped samples with random feature selection at each split [34]. This bagging strategy naturally handles nonlinear interactions while providing outlier resistance. However, RF’s voting-based aggregation tends to dampen extreme predictions, causing systematic peak underestimation. Hyperparameter tuning examined ensemble sizes (50, 100, 200 trees), maximum depths (None, 10, 20), and minimum samples (2, 5, 10) for splitting/leave. The optimal RF configuration (n_estimators = 200, max_depth = 20, min_samples_split = 5) balanced extreme value capture against spurious prediction avoidance.

XGB implements optimized gradient boosting with L1/L2 regularization penalties, intelligent tree pruning, and parallelized computation [35]. Sequential residual error correction adaptively allocates learning capacity to difficult samples including rare floods. Optimization explored learning rates (0.01, 0.1, 0.2), tree depths (3, 5, 7), estimator numbers (50, 100, 200), and subsample ratios (0.8, 1.0). The optimal XGB configuration (learning_rate = 0.1, max_depth = 5, n_estimators = 200, subsample = 0.8) was selected to maintain extreme event sensitivity while preventing overfitting.

All models were implemented using Python’s scikit-learn and PyTorch. Final models were independently evaluated on the held-out testing period (2005–2013) containing multiple severe typhoon events, providing rigorous assessment of generalization to unseen extreme flood scenarios.

### 2.5. Performance metrics

Model performance was assessed using four complementary metrics providing comprehensive evaluation of predictive accuracy and hydrological fidelity:


$$\begin{array}{c} RMSE\;=\;\sqrt{\frac{\text{1}}{n}\sum_{i=\text{1}}^{n}{\left(Q_{obs,i}-Q_{pred,i}\right)}^{\text{2}}} \end{array}$$ (2)

$$\begin{array}{c} MAE\;=\;\frac{\text{1}}{n}\sum_{i=\text{1}}^{n}\left|Q_{obs,i}-Q_{pred,i}\right| \end{array}$$ (3)

$$\begin{array}{c} NSE\;=\;\text{1}-\frac{\sum_{i=\text{1}}^{n}{\left(Q_{obs,i}-Q_{pred,i}\right)}^{\text{2}}}{\sum_{i=\text{1}}^{n}{\left(Q_{obs,i}-\overset{―}{Q_{obs}}\right)}^{\text{2}}} \end{array}$$ (4)

$$\begin{array}{c} KGE\;=\;\text{1}-\sqrt{{\left(r-\text{1}\right)}^{\text{2}}+{\left(\frac{\sigma_{pred}}{\sigma_{obs}}-\text{1}\right)}^{\text{2}}+{\left(\frac{\mu_{pred}}{\mu_{obs}}-\text{1}\right)}^{\text{2}}} \end{array}$$ (5)

where $Q_{obs,i}$ and $Q_{pred,i}$ denote observed and predicted streamflow at time *i*, *n* is the number of observations, $\overset{―}{Q_{obs}}$ represents mean observed flow, *r* is the correlation coefficient, *σ* denotes standard deviation, and *μ* represents mean values.

RMSE and MAE quantify absolute prediction errors in discharge units (m³/s), with RMSE assigning disproportionate weight to large deviations through its quadratic formulation. This sensitivity to outliers makes RMSE particularly valuable for assessing extreme flood event prediction accuracy, where peak magnitude errors carry the greatest operational consequence for early warning systems. NSE evaluates model performance relative to a climatological baseline defined by the long-term mean, with values approaching unity indicating substantial improvement over naive persistence forecasting. KGE provides a more comprehensive evaluation framework by decomposing overall performance into three independent components: linear correlation quantifying temporal pattern matching, bias ratio measuring systematic over- or underestimation tendencies, and variability ratio assessing the model’s capacity to reproduce observed flow magnitude ranges.

## 3. Results

### 3.1. Overall performance across three feature scenarios

To systematically evaluate the effectiveness of typhoon feature engineering, this study designed three contrasting scenarios: Baseline (containing only conventional hydrometeorological variables), With Typhoon (adding 8 original typhoon observational features), and Enhanced Typhoon (introducing 19 physics-driven derived features). Four machine learning models (LR, ANN, RF, XGB) were evaluated under a unified train-test split, with performance quantified using four standard hydrological metrics (NSE, RMSE, MAE, KGE).

**Fig 3:**
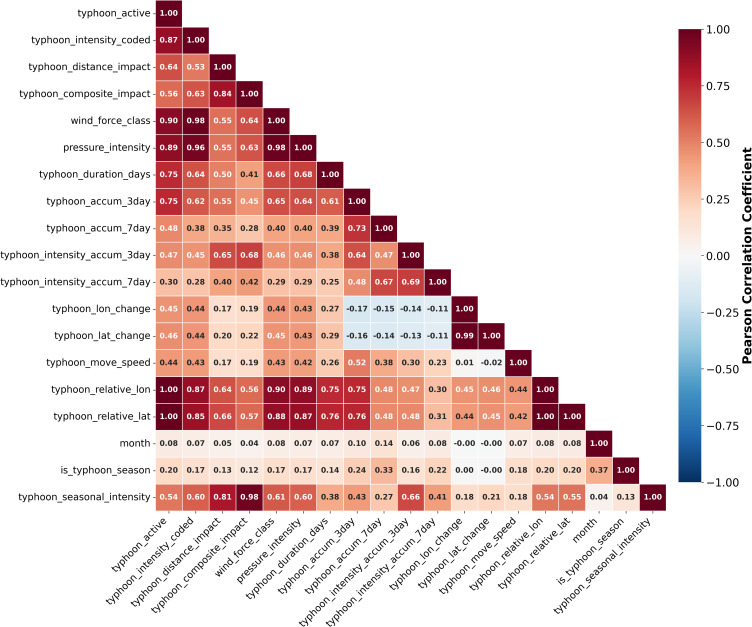
Correlation Matrix of 19 Typhoon Features.

To validate the necessity and redundancy of the 19 physics-informed typhoon features, a comprehensive sensitivity analysis using RFE and MI was conducted prior to model training and hyperparameter tuning. The cross-correlation analysis among the 19 features (Fig 3) revealed that while moderate to high correlations exist between certain feature pairs (e.g., 3-day and 7-day cumulative activity with r = 0.73, wind force level and pressure intensity with r = 0.98), most features maintain relatively independent information content with correlation coefficients below 0.6, suggesting limited redundancy in the overall feature set.

To examine whether dimensionality reduction could maintain model performance while reducing computational complexity, two feature selection strategies (RFE-based and MI-based selection) were compared as shown in Table 1. Both methods independently selected 50 critical features from the 133 time-lagged typhoon features (62.4% reduction), achieving 76% overlap in their selections and demonstrating high consistency in identifying key features such as typhoon composite impact, typhoon distance impact, and 7-day cumulative intensity. The RFE-selected feature set maintained comparable RMSE performance to the full feature set for ANN (173 m³/s vs 172 m³/s, a negligible 0.6% difference) while reducing inference time by 16.7% (0.005s vs 0.006s per sample). Similarly, the MI-selected features achieved RMSE of 175 m³/s (1.7% higher than full set) with marginally faster inference. These results demonstrate that while strategic feature selection can reduce computational cost with minimal accuracy loss, the full set of 19 physics-informed features collectively provides the optimal predictive performance. Consequently, all subsequent analyses retained the complete typhoon feature set to maximize model accuracy. From a computational feasibility perspective, all experiments were conducted on a 13th Gen Intel(R) Core(TM) i9-13900K processor at 3.00 GHz without GPU acceleration. Using the full feature set as the benchmark, the most computationally intensive training occurred with ANN, requiring 2.821 seconds per sample on average. However, inference speed (the critical metric for real-time operational systems) was faster across all models. Even the slowest model (RF) achieved inference at only 0.042 seconds per sample, well within the latency requirements for timely flood forecasting in engineering applications.

**Table 1:** Comparison of Sensitivity to Enhanced Typhoon Features and Training-Inference Performance Among Four Models (LR, ANN, RF and XGB).

	RMSE (Improvement, %)				Time (s/sample)	
	Baseline	With All Typhoon	With RFE Typhoon	With MI Typhoon	Train	Inference
**LR**	211	193 (+8.6%)	192 (+8.8%)	193 (+8.6%)	0.084	0.003
**ANN**	213	172 (+19.3%)	173 (+18.9%)	175 (+18.1%)	2.821	0.005
**RF**	318	357 (−12.1%)	343 (−7.9%)	347 (−9.1%)	0.596	0.042
**XGB**	258	256 (+0.6%)	256 (+0.6%)	255 (+0.9%)	1.113	0.014

**Fig 4:**
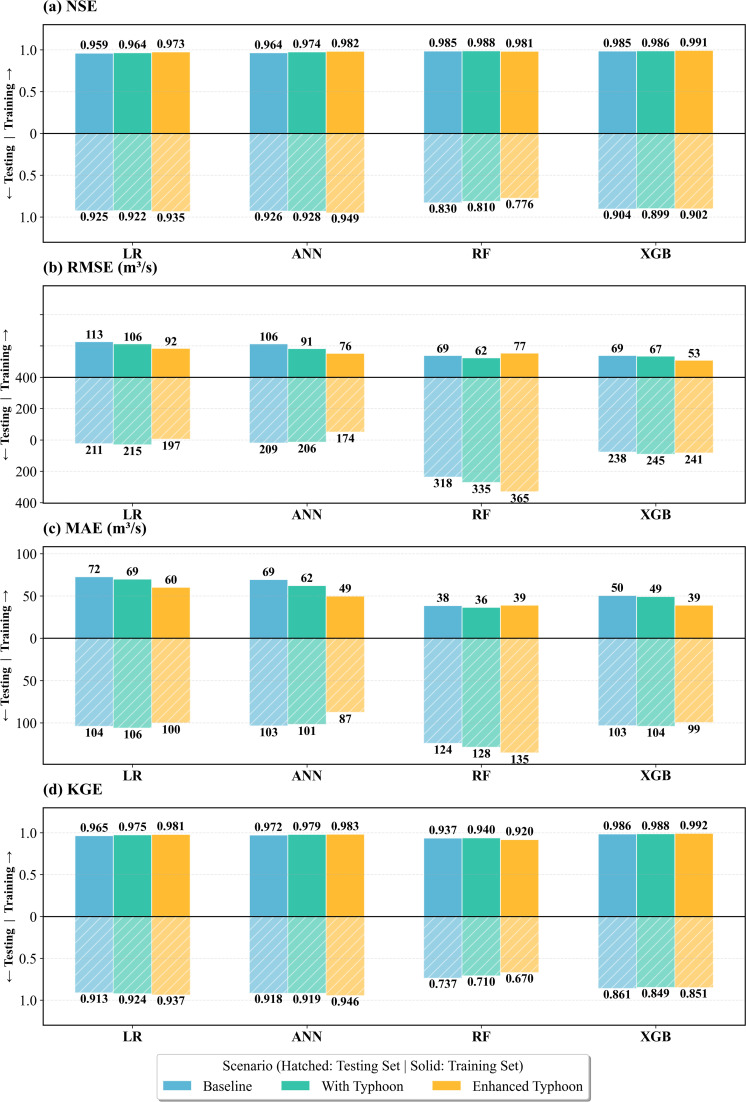
Overall model performance comparison across three feature engineering scenarios in the Boluo watershed.

Fig 4a-d demonstrates the systematic impact of typhoon feature engineering on model performance across both training and testing phases. The ANN-EnTY model exhibited optimal performance across all scenarios and showed the most positive response to feature engineering. In the training set, its NSE reached 0.982 and KGE reached 0.983, indicating a high degree of fit. This translated effectively to the testing set, where its NSE improved from 0.926 in the Baseline to 0.949 in Enhanced Typhoon (+2.5%), RMSE decreased from 209 m³/s to 174 m³/s (−16.7%), MAE reduced from 103 m³/s to 87 m³/s (−15.5%), and KGE increased from 0.918 to 0.946 (+3.1%). Notably, the improvement magnitude in RMSE significantly exceeded that in NSE, indicating that typhoon features primarily enhanced the prediction capability for extreme flows rather than overall average errors.

As a linear baseline method in testing set, LR achieved high performance (NSE = 0.925). Incorporating enhanced typhoon features further improved the NSE to 0.935, demonstrating a certain degree of performance enhancement. XGB demonstrated moderate but stable performance (NSE = 0.902), yet typhoon features yielded no further improvement. For RF, the NSE continuously decreased from 0.814 to 0.776, representing a reduction of 4.7%, while the RMSE deteriorated from 332 m³/s to 365 m³/s, corresponding to an increase of 9.9%. This counterintuitive phenomenon originates from RF’s sensitivity to high-dimensional feature spaces. The introduction of numerous typhoon features led RF to make suboptimal decisions during decision tree splitting, which aggravated the systematic underestimation of extreme values.

Comparing the With Typhoon and Enhanced Typhoon experiments quantifies the incremental value of feature engineering. Interestingly, direct use of original typhoon features failed to improve model accuracy, as all four models exhibited either unchanged or degraded performance. Only when employing engineered enhanced features did LR and ANN demonstrate improvement. The feature engineering design was based on several physical mechanisms, such as employing sigmoid functions to simulate nonlinear distance decay of typhoon influence, and constructing a comprehensive impact index to integrate multi-dimensional typhoon intensity information. This stems from ANN’s continuous activation functions being able to fully leverage smooth nonlinear relationships under physical constraints. In contrast, tree-based models (RF and XGB) showed limited or even negative response to enhanced features, because tree structures naturally implement piecewise linear approximations through split points, already capable of constructing similar nonlinear patterns from original features.

As a comprehensive metric, KGE simultaneously examines correlation (r), bias (β), and variability (γ). Fig 4d shows that typhoon features not only improved goodness-of-fit but also optimized the statistical properties of predictions. RF’s sharp decline in KGE (from 0.737 to 0.670) further confirms its complete failure under the enhanced feature scenario. Specifically, the model not only exhibited inaccurate peak prediction (characterized by a low β value) but also systematically underestimated variability (reflected by a low γ value).

Overall performance evaluation indicates that typhoon feature engineering provides statistically significant and practically meaningful improvements for runoff prediction, though effects are highly dependent on model architecture. ANN, with its powerful nonlinear expression capability and high sensitivity to physical features, performed optimally across all scenarios, establishing it as the preferred base model for this study. The close alignment between training and testing metrics for the ANN-EnTY model further demonstrates that the performance gains represent enhanced physical generalization rather than mere numerical fitting. Moreover, feature engineering must align with model characteristics and data structure. Blindly increasing feature dimensionality proves counterproductive through the curse of dimensionality when features approach sample size, introduction of noise from irrelevant variables that obscure genuine physical relationships, and increased overfitting risk from multicollinearity, particularly problematic for extreme events comprising limited training samples.

### 3.2. Seasonal performance variability

Seasonal analysis is crucial for understanding model performance under varying hydrological conditions. The test period was divided according to climatological standards into spring (March-May), summer (June-August), autumn (September-November), and winter (December-February), with summer and autumn representing the primary typhoon active periods in South China, while winter experiences virtually no typhoon activity.

**Fig 5:**
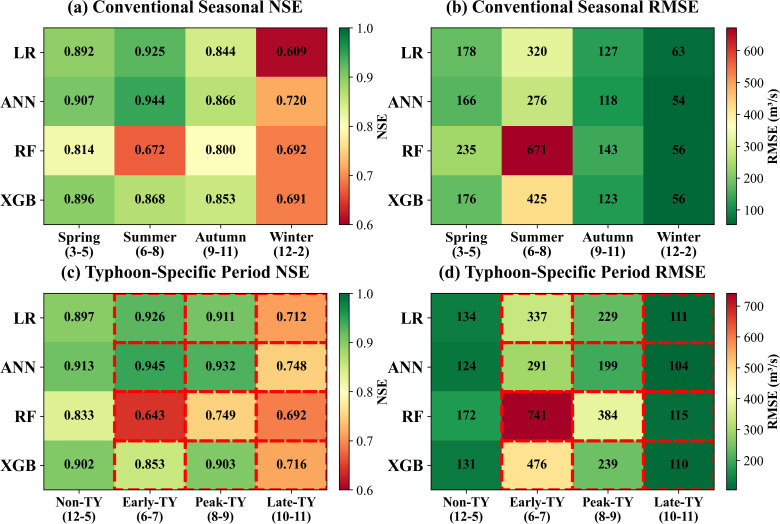
Seasonal performance comparison of four models under Enhanced Typhoon scenario.

Summer exhibited optimal overall performance alongside the greatest inter-model variability (Fig 5a). ANN-EnTY achieved the highest NSE (0.925), followed closely by LR-EnTY (0.892) and XGB-EnTY (0.896), while RF-EnTY experienced catastrophic collapse (NSE = 0.672, RMSE = 671 m³/s). RF-EnTY’s anomalous behavior stems from its sensitivity to high-frequency typhoon disturbances and high-dimensional feature spaces. Frequent summer typhoon events induced severe overfitting during decision tree splitting, causing the model to erroneously learn typhoon noise as predictive signals rather than meaningful hydrological patterns. RF-EnTY ‘s summer RMSE (671 m³/s) was 2.4 times that of ANN-EnTY (276 m³/s) and 2.9 times its own spring performance (235 m³/s), confirming systematic failure during typhoon-intensive periods (Fig 5b).

Autumn represents the season of most active and intense typhoon activity, with all models showing NSE decline compared to summer. ANN-EnTY ‘s NSE decreased to 0.866 but maintained optimality; XGB-EnTY followed closely (0.853), demonstrating the adaptability of boosting strategies to extreme samples; LR-EnTY declined to 0.844, exposing the limitations of its linear assumptions under extreme nonlinear scenarios; RF-EnTY showed slight recovery (0.800) but remained significantly inferior to other models. From the RMSE perspective, XGB-EnTY ‘s RMSE (425 m³/s) was anomalously high, 1.5 times that of ANN-EnTY (276 m³/s). In contrast, autumn RMSE for ANN-EnTY and RF-EnTY (118 and 143 m³/s, respectively) were significantly lower than summer values, a phenomenon attributable to reduced runoff magnitude in autumn.

Spring-winter comparisons revealed the critical role of typhoon activity. Spring, characterized by minimal typhoon activity, exhibited stable and convergent model performance. ANN-EnTY achieved NSE of 0.907, followed by XGB-EnTY (0.896), LR-EnTY (0.892), and RF-EnTY (0.814), with inter-model differences primarily reflecting inherent algorithmic capabilities rather than typhoon-handling capacity. Winter’s NSE dropped sharply (LR-EnTY = 0.609, XGB-EnTY = 0.691, RF-EnTY = 0.692, ANN-EnTY = 0.720), yet RMSE remained extremely low (ANN-EnTY = 54 m³/s, RF-EnTY = 56 m³/s, XGB-EnTY = 56 m³/s, LR-EnTY = 63 m³/s). This combination reflects that small runoff magnitude (averaging <500 m³/s) and gentle variations cause the NSE denominator (observed variance) to shrink significantly, making NSE extremely sensitive to small errors. Notably, winter RMSE converged across all models (54–63 m³/s), with inter-model differences nearly vanishing, indicating that under low-flow conditions, model architectural advantages are masked by the inherently low signal-to-noise ratio of the data.

The substantial seasonal performance differences are rooted in seasonal variability of hydrological processes. During summer and autumn, rainfall-runoff relationships dominate watershed response with relatively regular patterns (high signal-to-noise ratio), and model performance differences primarily manifest in typhoon event handling capabilities. In winter, runoff is controlled by mixed processes (baseflow, reservoir operations, agricultural withdrawals), with increased process complexity and lacking relevant features (low signal-to-noise ratio), causing all models to converge toward lower performance. The seasonal pattern of RMSE (highest in summer, lowest in winter) directly reflects seasonal variations in flow magnitude, highlighting that absolute error metrics require interpretation within the context of flow conditions when comparing across seasons.

To address the limitations of conventional four-season divisions that obscure typhoon-specific hydrological dynamics, the test period was reclassified into four typhoon-specific periods based on the actual timing of typhoon activity in South China: (1) Non-typhoon period (December -May), characterized by minimal typhoon influence and dominated by spring precipitation; (2) Early typhoon period (June-July), marking the onset of typhoon season with moderate frequency; (3) Peak typhoon period (August-September), representing the most active and intense typhoon months with the highest frequency of tropical cyclones; and (4) Late typhoon period (October-November), exhibiting declining but still significant typhoon activity. This typhoon-aligned temporal framework enables more precise evaluation of model sensitivity to varying levels of typhoon intensity and frequency (Fig5c-d). The typhoon-specific framework reveals nuanced performance patterns masked by conventional divisions. During the early typhoon period, ANN-EnTY maintained NSE of 0.945 with RMSE of 291 m³/s, demonstrating superior resilience to extreme typhoon events compared to the early typhoon period (NSE = 0.932, RMSE = 199 m³/s). RF-EnTY exhibited the most pronounced sensitivity to typhoon intensity, with NSE declining from 0.833 during non-typhoon periods to 0.749 during peak typhoon months. For LR-EnTY and ANN-EnTY, both early typhoon and peak typhoon periods exhibited higher NSE than the non-typhoon period, while the late typhoon period showed the poorest performance with NSE below 0.8. By October-November, weakened post-landfall typhoons produce degraded observational features (irregular wind fields, reduced pressure gradients), while the watershed transitions from saturated to drier states, fundamentally altering rainfall-runoff relationships that violate models’ training assumptions.

### 3.3. Performance across flow magnitude ranges

To deeply understand model predictive capability under varying hydrological conditions, test samples were stratified by observed flow percentiles into six magnitude categories: Very Low (<P25), Low (P25-P50), Medium (P50-P75), High (P75-P90), Very High (P90-P95), and Extreme (>P95). This stratified analysis evaluates model reliability during extreme flood events, which is essential for operational flood early warning systems.

**Fig 6:**
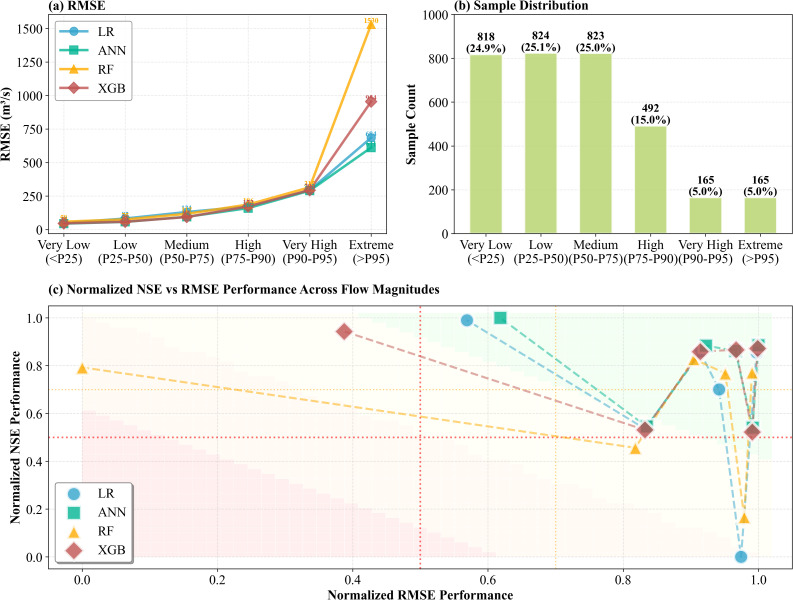
Model performance stratified by flow magnitude ranges. (a) RMSE across six magnitude categories showing exponential growth from low to extreme flows; (b) Sample distribution; (c) Normalized NSE versus normalized RMSE performance map. Normalization is performed within each magnitude category to enable cross-range.

All models exhibited monotonically increasing RMSE trends (Fig 6a), rising from 50–80 m³/s in Very Low to 600–1550 m³/s in Extreme ranges, representing 10–20-fold growth. This stems from: (1) high-flow events possess greater variability, naturally increasing prediction uncertainty; (2) Extreme samples are scarce (only 2%), leading to insufficient model training. Critically, inter-model differences are dramatically amplified in extreme ranges: in the Extreme category, RF’s RMSE (1550 m³/s) is 2.4 times that of ANN-EnTY (640 m³/s), with XGB-EnTY at 970 m³/s and LR-EnTY at 720 m³/s. RF-EnTY ‘s performance confirms its voting-averaging mechanism severely and systematically underestimates extreme values, while ANN-EnTY maintains optimal and most stable performance across the entire magnitude spectrum.

Fig 6b reveals test samples are highly concentrated in low-medium flow ranges: Very Low, Low, and Medium each comprise approximately 25% (818–824 samples), High represents 15% (492 samples), while Very High and Extreme each account for only 5% (165 samples). This severe class imbalance is an inherent characteristic of hydrological prediction, as extreme floods are rare yet consequential. Traditional overall performance metrics are predominantly influenced by normal flow conditions, potentially obscuring model performance during critical extreme events.

The two-dimensional perspective of Fig 6c (normalized NSE vs. normalized RMSE) reveals three critical patterns. First, high-performance clustering in low-medium flow ranges (upper-left green zone). Very Low through Medium ranges show all models concentrated in regions of high NSE (>0.95) and low RMSE (<0.5), with four dashed lines nearly overlapping, indicating model differences are insignificant under normal flow conditions. Second, performance divergence initiation in the High range (central transition zone). Normalized NSE decreases to 0.85–0.95 and normalized RMSE rises to 0.4–0.5, with models beginning to show visible separation. XGB-EnTY maintains highest NSE (0.95), followed closely by ANN-EnTY and LR-EnTY (0.93), while RF-EnTY drops significantly (0.53), marking its first collapse in extreme value handling capability. Third, performance collapse and model differentiation in extreme ranges (right-side region). Very High and Extreme ranges exhibit a “V-shaped” collapse pattern, with all models’ normalized NSE plummeting to 0.5–0.9 and normalized RMSE surging to 0.9–1.0. However, the severity of performance collapse exhibits strong model-dependent patterns. ANN-EnTY and XGB-EnTY maintained NSE of 0.85–0.88 in the Extreme range (solid lines), while LR-EnTY degraded to 0.70 and RF-EnTY collapsed to 0.15 (dashed lines).

As flow magnitude increases, normalized NSE declines while normalized RMSE rises. Yet ANN-EnTY demonstrates robustness advantages in extreme events by maintaining a relatively steep decline curve compared to RF-EnTY’s precipitous collapse. This two-dimensional map provides clear guidance for model selection. When flood early warning is the core objective, priority should be given to models maintaining high NSE in extreme flow ranges rather than models achieving highest overall NSE across all conditions.

### 3.4. Peak prediction and detection accuracy

The core of flood early warning systems lies in accurately predicting flood peaks and timely identifying flood events. This section focuses on model performance during flood periods (flow >P90), employing two complementary perspectives: peak prediction error distribution and flood detection confusion matrices.

**Fig 7:**
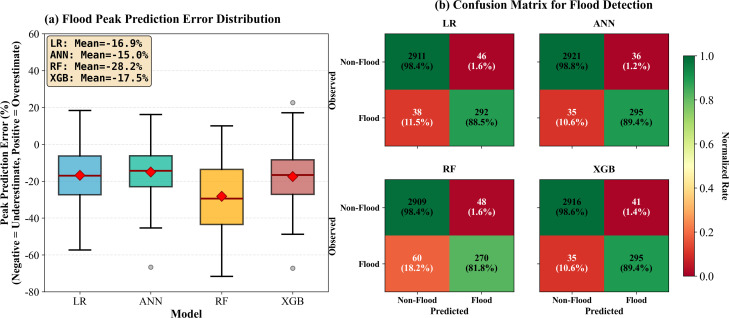
Flood period performance evaluation through peak prediction and event detection. (a) Box plots of peak prediction relative errors while negative values indicating underestimation. (b) Confusion matrices for binary flood detection (P90 threshold). Color intensity represents normalized rates, with green indicating correct classification and red indicating misclassification.

Fig 7a reveals that all models exhibit systematic underestimation tendencies, with negative mean relative errors: LR-EnTY (−16.9%), ANN-EnTY (−15.0%), RF-EnTY (−28.2%), and-EnTY XGB (−17.5%). This universal phenomenon stems from training sample class imbalance (flood samples comprise only 10%) and models’ conservative strategies. RF demonstrates the most severe underestimation, with median error reaching −28.2%, box range (−42% to −15%) significantly deviating from the zero line, and outliers extending to −70%. This confirms its voting mechanism’s systematic suppression of extreme values. In contrast, ANN-EnTY exhibits the smallest bias and most compact distribution: median −15.0%, box range (−25% to −5%), with upper quartile approaching the zero line. LR-EnTY and XGB-EnTY perform intermediately (−16.9% and −17.5%), though XGB-EnTY ‘s box range is slightly wider, indicating inferior consistency compared to ANN-EnTY. Even the optimal ANN-EnTY retains an average 15% underestimation, which may lead to inadequate warnings in practical applications.

Fig 7b’s confusion matrices evaluate binary classification performance using P90 as the threshold. ANN-EnTY demonstrates optimal comprehensive detection capability: true positive rate (recall) of 89.4% (295/330), false positive rate of merely 1.2% (36/2957), and false negative rate (miss rate) of 10.6% (35/330). This indicates ANN-EnTY can correctly identify nearly 90% of flood events while maintaining false alarm rates below 2%. Model differences primarily manifest in miss rates. RF-EnTY ‘s miss rate reaches 18.2% (60/330), nearly double that of ANN-EnTY, consistent with its systematic underestimation. XGB-EnTY and LR-EnTY both achieve 10.6% miss rates, matching ANN-EnTY, though XGB-EnTY ‘s false positive rate is slightly higher (1.4%). All models maintain extremely low false positive rates (1.2%−1.6%). In terms of F1-Score, ANN-EnTY achieves 0.934, significantly outperforming RF-EnTY (0.871) and marginally surpassing LR-EnTY /XGB-EnTY (0.915).

### 3.5. Flood events analysis

**Fig 8:**
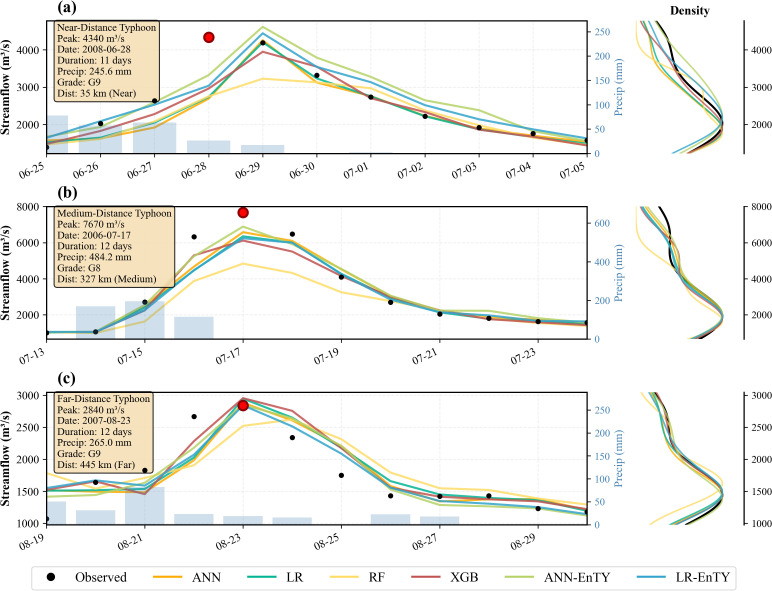
Model performance across three typhoon distance scenarios during extreme flood events: (a) Near-distance typhoon (55 km, June 28, 2008), (b) Medium-distance typhoon (327 km, July 17, 2006), and (c) Far-distance typhoon (445 km, August 23, 2007). Left panels show temporal evolution of observed (black dots) and predicted streamflow with precipitation (blue bars, right y-axis). Right panels display probability density distributions of streamflow predictions.

To evaluate model robustness under varying meteorological configurations, three extreme flood events were selected from the validation period based on the spatial proximity of the typhoon center to the watershed as illustrated in Fig 8. These events include a near-distance scenario (55 km occurring on June 28, 2008), a medium-distance scenario (327 km occurring on July 17, 2006), and a far-distance scenario (445 km occurring on August 23, 2007). Each event spans a complete hydrological cycle from the initial rising limb to the baseflow recession with total durations ranging between 11 and 13 days.

Fig 8a illustrates the hydrological response to a direct typhoon strike where the storm center passed within 35 km of the catchment and delivered 245.6 mm of cumulative precipitation. The discharge exhibited rapid intensification from a baseflow of approximately 1400 m³/s to a peak of 4340 m³/s within 3 days. Although all models exhibited a one-day phase lag in peak timing, the ANN-EnTY architecture produced a hydrograph that most closely mirrored the observed recession curve. Furthermore, the LR-EnTY model achieved a peak estimation of 4443 m³/s with a Relative Error (RE) of 2.3% which marked an improvement over the baseline LR model that recorded a peak of 4202 m³/s and an RE of 3.2%. Probability density distributions in the right panel indicate that the EnTY-enhanced models maintain tighter clustering around observed values throughout the event spectrum and suggest consistent improvements in predictive stability.

The most significant flood event within the study period occurred under medium-distance forcing at 327 km where peripheral circulation delivered 484.2 mm of rainfall over a 13-day duration as illustrated in Fig 8b. Discharge escalated rapidly from approximately 800 m³/s to an observed peak of 7670 m³/s within 4 days. In this high-magnitude event, ANN-EnTY achieved a predicted peak of 6890 m³/s which significantly outperformed the baseline ANN prediction of 6579 m³/s and resulted in an RE reduction of approximately 4%. Similarly, the LR-EnTY model reached a peak of 6348 m³/s whereas the standard LR model predicted 6242 m³/s and thereby yielded an improvement in RE of approximately 1%. The right-side density plot further confirms that the integration of typhoon-specific features bolsters model robustness across the entire flow spectrum during such extreme hydraulic loading conditions.

At a distance of 445 km, indirect precipitation mechanisms dominated flood generation with 265.0 mm of rainfall resulting in a moderate peak of 2840 m³/s as depicted in Fig 8c. ANN-EnTY maintained high accuracy with a negligible peak deviation of only −12 m³/s while the baseline ANN showed a larger deviation of 42 m³/s. Notably, the density distributions for all models show substantial overlap at this distance. This suggests that as the typhoon recedes, watershed memory and antecedent soil moisture conditions begin to override direct meteorological forcing and thereby diminish the marginal utility of typhoon-specific feature engineering.

The distance-stratified analysis reveals a gradient in the efficacy of typhoon-enhanced features. The improvement in peak prediction peaked at 4% under medium-distance forcing but moderated to 1% at near and far distances. Despite these variations, the enhanced models consistently outperformed their baseline counterparts across all scenarios and validated the robustness of physics-informed feature engineering for operational flood forecasting across diverse tropical cyclone configurations.

## 4. Discussion

### 4.1. Feature importance and hydrological interpretation

Understanding which features drive model predictions is critical for model trustworthiness and hydrological process comprehension. This study employs SHAP to interpret the ANN-EnTY model, revealing both aggregated feature importance patterns and individual prediction mechanisms.

**Fig 9:**
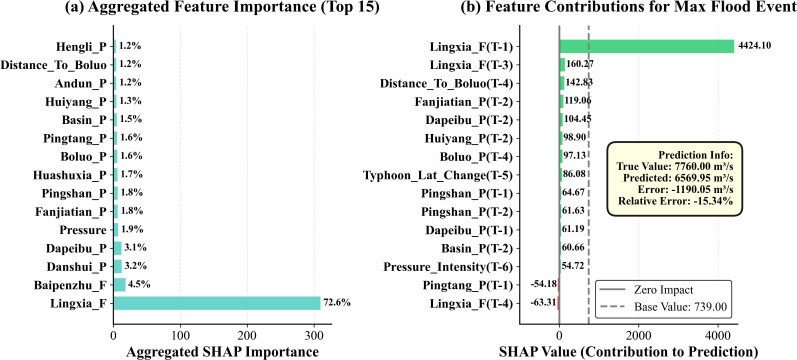
SHAP-based feature importance analysis for (a) aggregated global importance and (b) feature contributions during the maximum flood event.

Fig 9a demonstrates that Lingxia station flow dominates feature importance with an aggregated SHAP value of 72.6%, substantially exceeding all other features. Secondary important features include Baipenzhu flow (4.5%), Danshui rainfall (3.2%), and Dapeibu rainfall (3.1%), collectively accounting for approximately 11% of importance. As an upstream gauge located in close proximity to the Boluo outlet, Lingxia’s overwhelming dominance confirms that Boluo discharge is primarily governed by this tributary. Additionally, Baipenzhu, representing another upstream tributary subject to strong anthropogenic regulation, also exerts significant influence on Boluo as a secondary feature. Recent studies corroborate these findings, demonstrating that lagged flow features predominantly dominate machine learning-based runoff predictions [36], with reservoir storage temporal characteristics exhibiting particularly high predictive importance [37].

Typhoon-related features (e.g., typhoon pressure intensity) individually exhibit aggregated importance below 2%, appearing negligible when viewed in isolation. However, this aggregated perspective obscures the critical role of typhoon features during extreme events. Fig 9b presents the SHAP value decomposition for the maximum flood event on June 23, 2005, revealing the contribution patterns of typhoon features. During this extreme event, the T-1 flow at Lingxia station contributed 4424.10 m³/s, yet multiple typhoon features also demonstrated substantial contributions: T-4 typhoon distance to Boluo station (142.83 m³/s), T-3 typhoon latitude change (86.08 m³/s), and pressure intensity (54.72 m³/s). Critically, these typhoon features exhibit synergistic contributions, collectively explaining the sudden amplification effect of the flood peak. This finding aligns closely with cutting-edge research in extreme event prediction. Sezen & Šraj [12] noted that while machine learning models rely on dominant features for overall performance, they require integration of physically-constrained features to capture nonlinear processes during extreme events. Wang et al. [37] similarly emphasized the importance of meteorological features under extreme conditions, suggesting that model accuracy can be enhanced by incorporating relevant meteorological factors as key driving variables. The typhoon feature design in this study is precisely grounded in this rationale, providing the model with crucial physical drivers through physics-informed feature engineering to capture extreme events.

SHAP analysis further reveals the spatiotemporal dynamics of feature contributions. Fig 9b illustrates that features at different lag time steps exhibit differentiated contributions: short-term memory effects at T-1 (Lingxia flow), intermediate-term rainfall accumulation effects at T-2 (Fanjiatian, Dapeibu, Huiyang), and long-term typhoon effects at T-4, T-5, and T-6 (typhoon distance, latitude change, pressure intensity). This multi-timescale feature interaction reflects the physical processes of flood formation—typhoons trigger substantial moisture transport and low-pressure environments, subsequently inducing intensified precipitation, ultimately leading to rapid streamflow rises through channel routing. He et al. [38] observed similar multi-scale feature dynamics in multi-step runoff forecasting research, emphasizing the differentiated importance of time-series features across various prediction horizons.

Collectively, SHAP analysis not only validates the physical plausibility of the model [18,39] but also reveals the strategic value of feature engineering (typhoon features empowering extreme event prediction). This dual-mode pattern demonstrates that accurate extreme flood prediction requires the synergistic combination of dominant hydrological memory and modest but indispensable atmospheric forcing. The operational implication for flood early warning systems is that they must maintain high-quality upstream flow monitoring as the prediction backbone while strategically integrating physics-informed typhoon features to capture meteorological triggers that elevate routine flows into catastrophic extremes.

### 4.2. Physics-Driven typhoon feature engineering

Although typhoon features exhibit relatively low aggregated importance in Section 4.1, their critical role during extreme events underscores the strategic value of physics-driven feature engineering. This section dissects three core mechanisms of typhoon feature design: nonlinear distance decay functions, multi-dimensional feature correlation patterns, and cumulative lag effects, revealing why physically-constrained feature engineering significantly enhances extreme flood prediction capability.

#### 4.2.1. Nonlinear distance decay.

**Fig 10:**
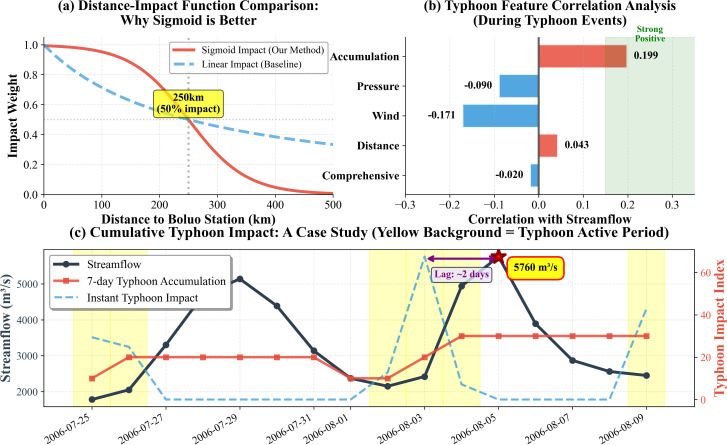
Physics-informed typhoon feature engineering for (a) sigmoid versus linear distance-impact function comparison, (b) feature-streamflow correlations during typhoon events, and (c) case study demonstrating cumulative typhoon impact on extreme flood generation.

Traditional approaches often employ simple reciprocal relationships (1/distance) to represent distance decay of typhoon influence, an assumption that ignores the physical nature of tropical cyclone impacts. Fig 10a compares the Sigmoid function proposed in this study with conventional linear methods. This design is grounded in three physical realities: (1) within the typhoon core region (<100 km), influence approaches saturation, maintaining high weights (>0.95); (2) in the transition zone (100–300 km), influence decays rapidly—the “S-shaped” characteristic of the Sigmoid function precisely captures this nonlinear process; (3) in the far-field region (>300 km), influence diminishes toward zero, with weights smoothly declining to near-zero values. In contrast, linear functions exhibit excessive decay in near-field regions (only 0.7 weight at 100 km) while maintaining insufficient decay in far-field regions (still 0.3 weight at 500 km), systematically deviating from physical reality. Recent research in tropical cyclone precipitation patterns supports this nonlinear spatial structure, demonstrating that precipitation intensity exhibits exponential decay with distance from storm centers [40].

#### 4.2.2. Differentiated correlation patterns of typhoon features.

Fig 10b presents Pearson correlation coefficients between five typhoon feature categories and Boluo station streamflow during typhoon-active periods. Results reveal significant differentiated correlation patterns among typhoon features: the typhoon accumulation index exhibits the strongest positive correlation (r = 0.199, p < 0.001), validating the cumulative effect hypothesis; typhoon distance influence shows the second-strongest positive correlation (r = 0.043), conforming to the physical expectation that closer distance yields greater impact; the comprehensive typhoon impact index displays weak negative correlation (r = −0.020), a counterintuitive phenomenon attributable to the instantaneous impact index primarily reflecting same-day typhoon intensity while streamflow response exhibits 1-3 day lags.

Notably, wind speed (r = −0.171) and pressure intensity (r = −0.090) both demonstrate negative correlations. This phenomenon’s physical mechanism lies in the fact that strong typhoons are typically accompanied by low-pressure centers and high wind speeds, but in watersheds distant from typhoon centers (Boluo typically >200 km from storm centers), high wind speeds primarily induce moisture transport rather than direct precipitation, and updrafts in low-pressure systems have significantly weakened at peripheral regions. Conversely, weakened typhoons more readily stagnate over watersheds, triggering floods through persistent precipitation. This weak typhoon, heavy rainfall paradox is widespread in South China. Recent hybrid modeling studies of rainfall-runoff relationships emphasize similar patterns, noting that machine learning models require integration of physically-constrained meteorological features to capture nonlinear processes during extreme events [41,42].

#### 4.2.3. Cumulative lag effects.

Typhoon impacts on runoff are not instantaneous responses but rather achieved through cumulative-lag mechanisms of spatiotemporal transfer. Fig 10c dissects the extreme flood event of July 17, 2006 (peak 5760 m³/s), revealing three temporal scale layers of physical processes. The first layer represents the typhoon-active period (yellow background region, August 3–6), during which the instantaneous typhoon impact index (blue dashed line) fluctuates dramatically with peaks reaching 60 units, reflecting rapid movement and intensity changes of the typhoon system. The second layer represents the precipitation accumulation period, where the 7-day cumulative typhoon impact index (red solid line) continues rising after typhoon passage, increasing from 10 units on August 3–30 units on August 5, characterizing watershed water storage increases and soil saturation enhancement from continuous precipitation. The third layer represents the runoff response period, with Boluo station streamflow (black solid line) peaking at 5760 m³/s on August 5, lagging approximately 2 days relative to typhoon center passage. This cascade process completely embodies the physical transfer chain from meteorological to hydrological scales [39].

The lag effect’s temporal scale (~2 days) closely matches watershed concentration time. The two instantaneous impact pulses (August 3 and August 9) correspond respectively to the typhoon’s initial influence and subsequent circulation effects, while the cumulative index’s sustained elevation (maintaining ~30 units during August 5–9) explains why streamflow recedes slowly after peaking—watershed storage release requires several days. This memory effect stems from antecedent precipitation-induced soil moisture increases and groundwater table elevation, rendering watersheds more sensitive to subsequent rainfall. Similar multi-scale feature dynamics have been observed in streamflow forecasting research employing deep learning, emphasizing differentiated importance of time-series features across various prediction horizons [43].

### 4.3. Limitations and future directions

Despite achieving promising results, this study exhibits several limitations requiring improvement. As shown in Section 3.4, all models systematically underestimate flood peaks. This primarily stems from training sample imbalance, since extreme events constitute only ~10% of the dataset [23]. Additionally, physical feature design limitations contribute to peak underestimation. Typhoon rainfall exhibits strong asymmetric distribution with maximum precipitation concentrated in the downshear-left or northeast quadrants [44–45], yet our distance-based features assume axisymmetric decay that inadequately captures this quadrant-dependent variability. Orographic amplification mechanisms, where terrain forces moist typhoon circulation upward and enhances precipitation on windward slopes [46], are not explicitly represented in our feature set. Sub-daily precipitation intensity fluctuations within typhoon eyewall and rainband passages, critical for capturing extreme flood peaks, are smoothed in our daily aggregation process. Future research should investigate quantile regression for direct extreme value modeling [47] or focal loss functions that automatically emphasize hard-to-predict samples. Moreover, multicollinearity among typhoon features (e.g., correlations between wind speed, pressure, and distance decay functions) may influence model performance. High multicollinearity can inflate variance in parameter estimates for linear models while having less impact on tree-based methods that naturally handle correlated features through recursive partitioning.

Although ANN achieved highest accuracy, its black-box nature restricts mechanistic understanding and provides no guarantee of water balance closure. Physics-Informed Machine Learning (PIML) offers promising solutions by embedding physical constraints into model architectures. Wang et al. [48] demonstrated that physics-encoded deep learning frameworks integrating process-based runoff models with neural networks achieved improved accuracy while maintaining mass conservation. To advance beyond current limitations, concrete implementation strategies should begin with incorporating physics-informed loss functions that penalize violations of mass balance and energy conservation principles, ensuring predictions remain physically plausible. Multi-task learning architectures can be employed to simultaneously predict streamflow and intermediate hydrological states such as soil moisture and baseflow components, thereby enforcing physical consistency across the modeling chain. Furthermore, ensemble approaches combining data-driven models with process-based models can leverage both predictive accuracy and physical interpretability. These hybrid architectures can encode water balance equations as soft constraints or integrate conceptual rainfall-runoff modules to bridge the gap between empirical performance and mechanistic understanding [12,49].

Section 4.1 revealed upstream flow dominance (72.6%) with relatively low rainfall importance, potentially reflecting inadequate input design. Current models employ only daily total rainfall, excluding critical variables like soil moisture and rainfall intensity. Wang et al. [50] showed that incorporating comprehensive soil moisture dynamics can improve the model for flood prediction, while Soltani et al. [51] demonstrated that assimilating remotely-sensed soil moisture substantially improved flood forecasting accuracy. To address these input limitations, feasible improvement pathways include integrating high-resolution satellite soil moisture products such as SMAP and Sentinel-1 with gauge observations through data fusion techniques to capture antecedent wetness conditions. Adopting hourly or 3-hourly temporal resolution instead of daily aggregation would resolve sub-daily rainfall intensity peaks that trigger flash flood responses during typhoon passages. Additionally, incorporating atmospheric variables such as vapor pressure deficit and boundary layer height can better represent evapotranspiration and infiltration processes that modulate watershed response during extreme typhoon events.

This study evaluated model performance using temporal validation with an independent test period, providing robust assessment of generalization to unseen meteorological conditions. However, spatial cross-validation through sub-basin division was not implemented, as the Boluo watershed’s compact configuration contains limited independent gauging stations with strong spatial correlation due to nested structure. Model behavior in upstream sub-basins or different climatic regions remains unclear, and spatial transferability represents an important dimension not addressed in this single-watershed study. Transfer learning research demonstrates that multi-basin joint training significantly improves target basin predictive accuracy [6]. Additionally, models lack uncertainty quantification. Prediction intervals are absent despite heteroscedastic errors increasing with flow magnitude (Section 3.4). Future research should develop ensemble prediction methods providing confidence intervals and advancing real-time forecasting system deployment [52].

## 5. Conclusions

This study developed and evaluated an integrated machine learning framework for typhoon-induced flood prediction in the Boluo watershed, South China, emphasizing the strategic value of physics-driven typhoon feature engineering for extreme event forecasting. Four machine learning models (LR, ANN, RF, XGB) were systematically compared across three feature scenarios: Baseline (conventional hydrometeorological variables), With Typhoon (original typhoon observations), and Enhanced Typhoon (19 physics-informed derived features). Results demonstrated that ANN-EnTY achieved superior accuracy across all scenarios (NSE = 0.949, RMSE = 174 m³/s), representing 3.1% improvement in KGE and 16.7 m³/s reduction in RMSE compared to baseline models. Critically, during the extreme flood events (peaks to 7760 m³/s), ANN-EnTY reduced peak prediction errors by approximately 4% relative to the best baseline models. Summer and autumn, representing primary typhoon-active periods, exhibited greatest inter-model variability. ANN-EnTY maintained optimal performance across all seasons (NSE = 0.720–0.925) Feature importance analysis via SHAP revealed that antecedent flow dominated predictions, with upstream Lingxia gauge exhibiting overwhelming importance (72.6%), while typhoon features, though contributing only approximately 2% in aggregated terms, played synergistic critical roles during extreme events. This dual-mode pattern of feature importance provides explicit mechanistic insights for operational flood early warning systems.

This study advances typhoon-flood prediction through physics-informed feature engineering, significantly improving extreme event forecasting. Future work should integrate physics-constrained neural networks, multi-source data fusion, and transfer learning to enhance model robustness under climate change.

## Supporting information

S1 File Map source. Zip archive containing the source files used to generate the maps in this study.
(ZIP)

## References

[1] Daramola S, Muñoz DF, Muñoz P, Saksena S, Irish J. Predicting the Evolution of Extreme Water Levels With Long Short‐Term Memory Station‐Based Approximated Models and Transfer Learning Techniques. Water Resources Research. 2025;61(3). doi: 10.1029/2024wr039054

[2] Slater L, Blougouras G, Deng L, Deng Q, Ford E, Hoek van Dijke A, et al. Challenges and opportunities of ML and explainable AI in large-sample hydrology. Philos Trans A Math Phys Eng Sci. 2025;383(2302):20240287. doi: 10.1098/rsta.2024.0287 40739919 PMC12334205

[3] Niu Z, Huang W, Zhang L, Deng L, Wang H, Yang Y, et al. Improving Typhoon Predictions by Integrating Data‐Driven Machine Learning Model With Physics Model Based on the Spectral Nudging and Data Assimilation. Earth and Space Science. 2025;12(2). doi: 10.1029/2024ea003952

[4] Mesias CG, Bagtasa G. AI‐Based Tropical Cyclone Rainfall Forecasting in the Philippines Using Machine Learning. Meteorological Applications. 2025;32(4). doi: 10.1002/met.70083

[5] Yifru BA, Lim KJ, Bae JH, Park W, Lee S. A hybrid deep learning approach for streamflow prediction utilizing watershed memory and process-based modeling. Hydrology Research. 2024;55(4):498–518. doi: 10.2166/nh.2024.016

[6] Solanki H, Vegad U, Kushwaha A, Mishra V. Improving Streamflow Prediction Using Multiple Hydrological Models and Machine Learning Methods. Water Resources Research. 2025;61(1). doi: 10.1029/2024wr038192

[7] Frame JM, Kratzert F, Gupta HV, Ullrich P, Nearing GS. On strictly enforced mass conservation constraints for modelling the Rainfall‐Runoff process. Hydrological Processes. 2023;37(3). doi: 10.1002/hyp.14847

[8] AlDahoul N, Ahmed AN, Allawi MF, Sherif M, Sefelnasr A, Chau KW. A comparison of machine learning models for suspended sediment load classification. Engineering Applications of Computational Fluid Mechanics. 2022;16(1):1211–32.

[9] Sharma PJ, Patel PL, Jothiprakash V. Impact of rainfall variability and anthropogenic activities on streamflow changes and water stress conditions across Tapi Basin in India. Sci Total Environ. 2019;687:885–97. doi: 10.1016/j.scitotenv.2019.06.097 31412492

[10] Bhasme P, Vagadiya J, Bhatia U. Enhancing predictive skills in physically-consistent way: Physics Informed Machine Learning for hydrological processes. Journal of Hydrology. 2022;615:128618. doi: 10.1016/j.jhydrol.2022.128618

[11] Adera S, Bellugi D, Dhakal A, Larsen L. Streamflow Prediction at the Intersection of Physics and Machine Learning: A Case Study of Two Mediterranean‐Climate Watersheds. Water Resources Research. 2024;60(7). doi: 10.1029/2023wr035790

[12] Sezen C, Šraj M. Improving the simulations of the hydrological model in the karst catchment by integrating the conceptual model with machine learning models. Sci Total Environ. 2024;926:171684. doi: 10.1016/j.scitotenv.2024.171684 38508277

[13] Wang Z, Xu N, Bao X, Wu J, Cui X. Spatio-temporal deep learning model for accurate streamflow prediction with multi-source data fusion. Environmental Modelling & Software. 2024;178:106091. doi: 10.1016/j.envsoft.2024.106091

[14] Ougahi JH, Rowan JS. Enhanced streamflow forecasting using hybrid modelling integrating glacio-hydrological outputs, deep learning and wavelet transformation. Sci Rep. 2025;15(1):2762. doi: 10.1038/s41598-025-87187-1 39843529 PMC11754805

[15] Mosavi A, Ozturk P, Chau K. Flood Prediction Using Machine Learning Models: Literature Review. Water. 2018;10(11):1536. doi: 10.3390/w10111536

[16] Sun Y, Song Y, Qiao B, Li B. Distributed Typhoon Track Prediction Based on Complex Features and Multitask Learning. Complexity. 2021;2021(1). doi: 10.1155/2021/5661292

[17] Chen R, Zhang W, Wang X. Machine Learning in Tropical Cyclone Forecast Modeling: A Review. Atmosphere. 2020;11(7):676. doi: 10.3390/atmos11070676

[18] Lin G-F, Jhong B-C. A real-time forecasting model for the spatial distribution of typhoon rainfall. Journal of Hydrology. 2015;521:302–13. doi: 10.1016/j.jhydrol.2014.12.009

[19] Humphrey GB, Gibbs MS, Dandy GC, Maier HR. A hybrid approach to monthly streamflow forecasting: Integrating hydrological model outputs into a Bayesian artificial neural network. Journal of Hydrology. 2016;540:623–40. doi: 10.1016/j.jhydrol.2016.06.026

[20] Kratzert F, Klotz D, Shalev G, Klambauer G, Hochreiter S, Nearing G. Towards learning universal, regional, and local hydrological behaviors via machine learning applied to large-sample datasets. Hydrol Earth Syst Sci. 2019;23(12):5089–110. doi: 10.5194/hess-23-5089-2019

[21] Nevo S, Morin E, Gerzi Rosenthal A, Metzger A, Barshai C, Weitzner D, et al. Flood forecasting with machine learning models in an operational framework. Hydrol Earth Syst Sci. 2022;26(15):4013–32. doi: 10.5194/hess-26-4013-2022

[22] Gauch M, Kratzert F, Klotz D, Nearing G, Lin J, Hochreiter S. Rainfall–runoff prediction at multiple timescales with a single Long Short-Term Memory network. Hydrol Earth Syst Sci. 2021;25(4):2045–62. doi: 10.5194/hess-25-2045-2021

[23] Xie T, Hu C, Liu C, Li W, Niu C, Li R. Study on long short-term memory based on vector direction of flood process for flood forecasting. Sci Rep. 2024;14(1):21446. doi: 10.1038/s41598-024-72205-5 39271901 PMC11399255

[24] Pu J, Mu M, Feng J, Zhong X, Li H. A fast physics-based perturbation generator of machine learning weather model for efficient ensemble forecasts of tropical cyclone track. npj Clim Atmos Sci. 2025;8(1). doi: 10.1038/s41612-025-01009-9

[25] Yu Q, Liu C, Li R, Lu Z, Bai Y, Li W, et al. Research on a hybrid model for flood probability prediction based on time convolutional network and particle swarm optimization algorithm. Sci Rep. 2025;15(1):6870. doi: 10.1038/s41598-024-80100-2 40011464 PMC11865522

[26] Ibebuchi CC, Abu I-O. Probabilistic flood susceptibility mapping using explainable AI for the Western United States. Environ Res Commun. 2025;7(10):105008. doi: 10.1088/2515-7620/ae0c5c

[27] Huang H, Wang Z, Liao Y, Gao W, Lai C, Wu X, et al. Improving the explainability of CNN-LSTM-based flood prediction with integrating SHAP technique. Ecological Informatics. 2024;84:102904. doi: 10.1016/j.ecoinf.2024.102904

[28] Xu Y, Lin K, Hu C, Chen X, Zhang J, Xiao M, et al. Uncovering the Dynamic Drivers of Floods Through Interpretable Deep Learning. Earth’s Future. 2024;12(10). doi: 10.1029/2024ef004751

[29] Lamane H, Ech-chatbi A, Elyoussfi H, El Mostafa B, Chao J, Chebak A. Interpreting machine learning models based on SHAP values in predicting suspended sediment concentration. International Journal of Sediment Research. 2025;40(1):91–107.

[30] Fryer D, Strumke I, Nguyen H. Shapley Values for Feature Selection: The Good, the Bad, and the Axioms. IEEE Access. 2021;9:144352–60. doi: 10.1109/access.2021.3119110

[31] Kumar S, Ravi V. Explainable artificial intelligence (XAI) for interpreting predictive models and key variables in flood susceptibility. Journal of Hydrology: Regional Studies. 2024;57:102202.

[32] Hastie T, Tibshirani R, Friedman J. The elements of statistical learning: Data mining, inference, and prediction. 2 ed. Springer. 2009.

[33] Goodfellow I, Bengio Y, Courville A. Deep Learning. MIT Press. 2016.

[34] Breiman L. Random Forests. Machine Learning. 2001;45(1):5–32. doi: 10.1023/a:1010933404324

[35] Chen T, Guestrin C. XGBoost: A scalable tree boosting system. In: Proceedings of the 22nd ACM SIGKDD International Conference on Knowledge Discovery and Data Mining, 2016. 785–94.

[36] Zhang Z, Wang D, Mei Y, Zhu J, Xiao X. Developing an explainable deep learning module based on the LSTM framework for flood prediction. Front Water. 2025;7. doi: 10.3389/frwa.2025.1562842

[37] Wang S, Peng H. Multiple spatio-temporal scale runoff forecasting and driving mechanism exploration by K-means optimized XGBoost and SHAP. Journal of Hydrology. 2024;630:130650. doi: 10.1016/j.jhydrol.2024.130650

[38] He M, Xu X, Wu S, Kang C, Huang B. Multi-step ahead forecasting of daily streamflow based on the transform-based deep learning model under different scenarios. Sci Rep. 2025;15(1):5451. doi: 10.1038/s41598-025-89837-w 39953056 PMC11829044

[39] Ley A, Bormann H, Casper M. Linking explainable artificial intelligence and soil moisture dynamics in a machine learning streamflow model. Hydrology Research. 2024;55(6):613–27. doi: 10.2166/nh.2024.003

[40] Qin L, Zhu L, Liu B, Li Z, Tian Y, Mitchell G, et al. Global expansion of tropical cyclone precipitation footprint. Nat Commun. 2024;15(1):4824. doi: 10.1038/s41467-024-49115-1 38844448 PMC11156673

[41] Feng D, Beck H, Lawson K, Shen C. The suitability of differentiable, physics-informed machine learning hydrologic models for ungauged regions and climate change impact assessment. Hydrol Earth Syst Sci. 2023;27(12):2357–73. doi: 10.5194/hess-27-2357-2023

[42] Zhong L, Lei H, Gao B. Developing a Physics‐Informed Deep Learning Model to Simulate Runoff Response to Climate Change in Alpine Catchments. Water Resources Research. 2023;59(6). doi: 10.1029/2022wr034118

[43] Fan M, Zhang L, Liu S, Yang T, Lu D. Investigation of hydrometeorological influences on reservoir releases using explainable machine learning methods. Front Water. 2023;5. doi: 10.3389/frwa.2023.1112970

[44] Xu W, Jiang H, Kang X. Rainfall asymmetries of tropical cyclones prior to, during, and after making landfall in South China and Southeast United States. Atmospheric Research. 2014;139:18–26. doi: 10.1016/j.atmosres.2013.12.015

[45] Yu Z, Wang Y, Xu H. Observed Rainfall Asymmetry in Tropical Cyclones Making Landfall over China. Journal of Applied Meteorology and Climatology. 2015;54(1):117–36. doi: 10.1175/jamc-d-13-0359.1

[46] Agyakwah W, Lin Y-L. Generation and enhancement mechanisms for extreme orographic rainfall associated with Typhoon Morakot (2009) over the Central Mountain Range of Taiwan. Atmospheric Research. 2021;247:105160. doi: 10.1016/j.atmosres.2020.105160

[47] Nearing G, Cohen D, Dube V, Gauch M, Gilon O, Harrigan S, et al. Global prediction of extreme floods in ungauged watersheds. Nature. 2024;627(8004):559–63. doi: 10.1038/s41586-024-07145-1 38509278 PMC10954541

[48] Wang C, Jiang S, Zheng Y, Han F, Kumar R, Rakovec O, et al. Distributed Hydrological Modeling With Physics‐Encoded Deep Learning: A General Framework and Its Application in the Amazon. Water Resources Research. 2024;60(4). doi: 10.1029/2023wr036170

[49] Ali ASA, Jazaei F, Clement TP, Waldron B. Physics-informed neural networks in groundwater flow modeling: Advantages and future directions. Groundwater for Sustainable Development. 2024;25:101172. doi: 10.1016/j.gsd.2024.101172

[50] Wang Y, Shi L, Hu Y, Hu X, Song W, Wang L. A comprehensive study of deep learning for soil moisture prediction. Hydrol Earth Syst Sci. 2024;28(4):917–43. doi: 10.5194/hess-28-917-2024

[51] Soltani SS, Belleflamme A, Goergen K, Kollet S. Improving Real-Time Flood Forecasting: Probabilistic Validation of Assimilated Remotely-Sensed Soil Moisture Data. Earth Syst Environ. 2025;10(2):1961–86. doi: 10.1007/s41748-025-00726-8

[52] Hadi FAA, Mohd Sidek L, Ahmed Salih GH, Basri H, Sammen SSh, Mohd Dom N, et al. Machine learning techniques for flood forecasting. Journal of Hydroinformatics. 2024;26(4):779–99. doi: 10.2166/hydro.2024.208

